# Evaluation of the Predictive Value of Urine Leukocyte Esterase Test in *Chlamydia trachomatis* and *Neisseria gonorrhoeae* Infection Among Males Attending HIV/STI Clinics in Guangdong Province, China

**DOI:** 10.3389/fmed.2022.858165

**Published:** 2022-03-21

**Authors:** Xueying Yu, Peizhen Zhao, Zhida Mai, Qingqing Xu, Wentao Chen, Zhiqiao Wu, Xiaojuan Luo, Zhizhou Wu, Xiaofeng Liu, Qian Wu, Heping Zheng, Yaohua Xue

**Affiliations:** ^1^Department of Clinical Laboratory, Dermatology Hospital, Southern Medical University, Guangzhou, China; ^2^Department of Clinical Laboratory, Shanghai Fourth People's Hospital Affiliated to Tongji University School of Medicine, Shanghai, China; ^3^Department of Clinical Laboratory, The Sixth People's Hospital of Dongguan, Dongguan, China; ^4^Department of Clinical Laboratory, The First People's Hospital of Foshan, Foshan, China; ^5^Department of Clinical Laboratory, Dermatology Hospital of Jiangmen, Jiangmen, China; ^6^Department of Clinical Laboratory, The Third People's Hospital of Zhuhai, Zhuhai, China

**Keywords:** *Chlamydia trachomatis*, *Neisseria gonorrhoeae*, leukocyte esterase test, nucleic acid amplification test, cost-effective, prevalence

## Abstract

Leukocyte esterase test (LET) detection is a simple and inexpensive test performed by urinalysis. This study investigated the predictive value of LET for *Chlamydia trachomatis* (CT) and *Neisseria gonorrhoeae* (NG) infection among men attending HIV and sexually transmitted infection (HIV/STI) clinics in Guangdong Province, China. A total of 5,509 urine samples were collected from HIV and sexually transmitted infection clinics in Guangdong Province between 2017 and 2019. Specimens from 5,464 males were tested by both LET and nucleic acid amplification test (NAAT). Of 5,464 males, 497 (9.1%) tested positive for CT or NG by NAAT, with respective prevalence rates of 6.4% (95% confidence interval [95% CI]: 5.8–7.1%) and 3.8% (95% CI: 3.3–4.3%), including 1.2% (95% CI: 0.9–1.4%) co-infected. Compared to the HIV-negative individuals, individuals living with HIV tend to have a higher prevalence of CT, NG and co-infection with CT and NG. The LET sensitivity, specificity, positive predictive value (PPV), and negative predictive value (NPV) for CT were 46.4% (95% CI: 41.2–51.7%), 92.0% (95% CI: 91.2–92.7%), 28.4% (95% CI: 24.8–32.1%), and 96.1% (95% CI: 95.6–96.7%), respectively. The LET sensitivity, specificity, PPV, and NPV for NG were 68.4% (95% CI: 62.1–74.7%), 91.8% (95% CI: 91.1–92.6%), 25.0% (95% CI: 21.4–28.5%), and 98.7% (95% CI: 98.3–99%), respectively. Compared to the HIV-negative individuals, higher sensitivity and specificity were observed for HIV-positive individuals, but there was no statistical difference. The incremental cost-effectiveness ratio (ICER) using economic costs per additional person CT positive and NG positive was –$238.74 and –$145.60 compared with LET positive, respectively. LET is a cost-effective test and will be valuable for predicting CT and NG infection, which is highly prevalent in low- and middle-income countries.

## Introduction

*Chlamydia trachomatis* (CT) and *Neisseria gonorrhoeae* (NG) are the two most prevalent bacterial sexually transmitted infections (1). An estimated 376 million people globally are newly infected with STIs each year, including 127 million newly infected with CT and 87 million with NG, and this number continues to increase yearly (2). Undetected infections that go untreated can have serious complications including pelvic inflammatory disease, infertility, epididymitis, male infertility, and the further transmission of the infections (3, 4). Moreover, sexually transmitted infections (STIs) such as CT and NG infection might facilitate HIV-1 transmission, either directly through their effect on viral replication or indirectly through stimulating leukocyte activity and causing HIV shedding and/or disruption of the mucosal barrier (5, 6). Therefore, the detection of CT and NG in people with HIV is a priority because of the potential for these infections to enhance HIV transmission (7, 8).

CT and NG infections can be verified using multiple methods including microscopy, culture, enzyme immunoassay (EIA), direct fluorescent antibody (DFA) test, and nucleic acid amplification test (NAAT), which have been developed for screening and diagnosis (9). The NAAT is the most sensitive and specific assay for detecting CT and NG infections. As a non-invasive specimen, urine is considered an acceptable and recommended sampling strategy for men. NAAT has been used for CT or NG screening using urine sample in developed countries (10). However, urine samples have not been commonly used for CT and NG detection in developing countries and remote settings due to laboratory requirements and the high cost (11), which have limited its scaling-up in low- and middle-income countries.

The leukocyte esterase test (LET) is an essential component of routine urine tests. This non-specific, rapid dipstick assay is used to detect the presence of an esterase enzyme produced by polymorphonuclear leukocytes (white blood cells) in urine. The urine test is an indispensable preliminary examination performed during the diagnosis of many diseases (12, 13). Leukocyte esterase detection during a routine urine examination is considered to be moderately sensitive and specific for detecting the presence of urinary tract infections (14), and the LET can also be used to screen for CT and NG infection (15, 16).

Previous studies reported LET sensitivity ranging from 45 to 100% for the detection of urogenital CT infections in men, with specificity ranging from 78% to 96% (16, 17). Compared with the NAAT results for NG and CT detection, LET alone showed sensitivities of 66.7% and 60% and specificities of 90.7% and 94.7%, respectively, among Central Australian Aboriginal individuals (18). The variety of outcomes can be explained by differences in settings, patient groups, experimental test thresholds, and reference tests. However, no study has evaluated LET performance for CT and NG testing in the Chinese context. To fill this gap, the aims of our study were to investigate the prevalence of CT and NG infections among men attending HIV/sexually transmitted infection clinics in Guangdong Province of China from 2017 to 2019 and to evaluate the diagnostic performance of the LET for detecting urogenital CT and NG infections.

## Materials and Methods

### Study Sites and Patients

This study was conducted retrospectively on routinely collected data gathered from four geographic areas of Guangdong Province, which included the Pearl River Delta and the western, northern, and eastern regions of the province. The participants were men attending HIV/sexually transmitted infection clinics from a governmental sentinel surveillance network comprising 10 HIV/STI clinics in 10 cities in Guangdong Province: Maoming, Jiangmen, Foshan, Dongguan, Shaoguan, Zhanjiang, Shantou, Qingyuan, Zhuhai, and Chaozhou. Dongguan, Foshan, Zhuhai and Jiangmen located in the pearl river area which was more developed area. Eastern region (Shantou, Chaozhou), Western region (Maoming, Zhanjiang) and Northern region (Shaoguan, Qingyuan) are the underdeveloped regions in Guangdong Province, the economic level is far behind the Pearl River Delta region. A serial cross-sectional survey was routinely conducted in these sites among males ≥14 years old attending HIV/STI clinics between April and June each year. After providing verbal informed consent, those who agreed to participate in the study were referred to complete a questionnaire including demographic, behavioral, and clinical information with the help of a research assistant. Urine samples were collected from all eligible participants for subsequent testing. The protocol and questionnaire had been approved by the ethics committee of the Dermatology Hospital of Southern Medical University.

### Sample Size

The primary outcome of this study was the sensitivity of CT and NG diagnosis. We assume that the sensitivity of CT and NG diagnosis can both reach 80%. According to previous studies, the prevalence of CT and NG among males attending HIV/STI clinics in Guangdong Province was 8.6% (19) and 2.7% (20), respectively. We applied Two-sided Confidence Intervals (CI) for One Sensitivity method to estimate a sample size of 1,407 for CT and 4,482 for NG to produce a two-sided 95% CI and a width of 0.15 using PASS15.0. In order to ensure the power of the study, we should include at least 4,482 participants.

### Specimen Handling

Sample collection occurred between 2017 and 2019. All male patients were asked to provide a 20–30 mL midstream urine sample that was divided into two portions: one was tested immediately by LET (ARKRAY, Shiga, Japan) at local clinics, and the other was sent to the Dermatology Hospital of Southern Medical University, where NAAT was performed to detect CT and NG. Urine samples were transported under appropriate temperature control conditions to the study laboratory for processing and testing. Samples for NAAT were stored at 4°C until processing.

### Urine LET

LET was performed during routine urinalysis at local clinics. Specimens were subjected to LET using the dry chemical method. Either a positive or negative result was reported for every specimen. The level of enzymatic activity was classified as 0, 1+, 2+, or 3+, as indicated by the manufacturer's colorimetric chart. The clinic guidelines considered a urine sample to be LET positive if the enzymatic activity was 1+, 2+, or 3+. The cost for each LET was approximately $1.30.

### Detection of CT and NG

DNA in male urine samples was extracted and detected for CT and NG using the Roche cobas® 4800 CT/NG (Roche Diagnostics, Mannheim, Germany). The cobas 4800 system is a diagnostic assay that uses an automated workstation to isolate nucleic acids from clinical specimens and includes a real-time instrument for the detection of CT and NG. Experiments were performed according to the manufacturer's instructions. The cost for each test of CT or NG was approximately $11.00.

### Statistical Analyses

Data were analyzed using SPSS Version 22 (IBM Corp., Armonk, NY, USA). Categorical parameters are presented as the number (percentage) of patients. The Chi-square test was used to compare the prevalence of CT, NG and coinfection among different characteristics. The sensitivity, specificity, positive predictive value (PPV), and negative predictive value (NPV), with 95% confidence intervals (95% CIs), were calculated for LET, as compared to the testing results of NAAT. We estimated the total unit and incremental unit cost for each method. For all analyses performed in this study, the results were considered significant at *P* < 0.05.

## Results

### Study Population

Overall, 5,509 urine samples were collected from men attending HIV/STI clinics between April and July from 2017 to 2019 in 10 cities of Guangdong Province, China. Of these, 5,464 urine samples had valid NAAT and LET results (Table 1). The median age of participants was 37 years (range: 14–87 years). The HIV prevalence was 0.7% (37/5,464, 95% CI:0.5–0.9%). The majority of participants were less than 35 years old (44.5%, 2,434/5,464) and from the Pearl River Delta (41.9%, 2,292/5,464).

**Table 1:** Characteristics of the study population and prevalence of *Chlamydia* and *Gonorrhoeae* infections in Guangdong, China, 2017–2019 (*N* = 5,464).

**Characteristics**	**Total**	**CT-positive**		***P***	**NG-positive**		***P***	**CT & NG-positive**		***P***
		**Number % (95% CI)**			**Number % (95% CI)**			**Number % (95% CI)**		
Total	5,464	351	6.4 (5.8–7.1)		209	3.8 (3.3–4.3)		63	1.2 (0.9–1.4)	
Year				0.146			0.808			0.412
2017	1,958	139	7.1 (6.0–8.2)		71	3.6 (2.8–4.5)		19	1.0 (0.5–1.4)	
2018	2,243	127	5.7 (4.7–6.6)		90	4.0 (3.2–4.8)		31	1.4 (0.9–1.9)	
2019	1,263	85	6.7 (5.3–8.1)		48	3.8 (2.7–4.9)		13	1.0 (0.5–1.6)	
Age				0.001			0.289			0.572
≤18	102	8	7.8 (2.6–13.1)		8	7.8 (2.6–13.1)		3	2.9 (0.0–6.2)	
19–25	715	60	8.4 (6.4–10.4)		26	3.6 (2.3–5.0)		6	0.8 (0.2–1.5)	
26–35	1,617	116	7.2 (5.9–8.4)		71	4.4 (3.4–5.4)		21	1.3 (0.7–1.9)	
36–45	1,335	85	6.4 (5.1–7.7)		47	3.5 (2.5–4.5)		14	1.0 (0.5–1.6)	
46–55	926	59	6.4 (4.8–7.9)		30	3.2 (2.1–4.4)		11	1.2 (0.5–1.9)	
56–65	453	17	3.8 (2.0–5.5)		16	3.5 (1.8–5.2)		6	1.3 (0.3–2.4)	
>65	316	6	1.9 (0.4–3.4)		11	3.5 (1.5–5.5)		2	0.6 (0.0–1.5)	
Location				0.746			0.030			0.354
Pearl River Delta	2,292	141	6.2 (5.2–7.1)		67	2.9 (2.2–3.6)		20	0.9 (0.5–1.3)	
Eastern region	1,391	98	7.0 (5.7–8.4)		60	4.3 (3.2–5.4)		18	1.3 (0.7–1.9)	
Western region	890	56	6.3 (4.7–7.9)		40	4.5 (3.1–5.9)		14	1.6 (0.8–2.4)	
Northern region	891	56	6.3 (4.7–7.9)		42	4.7 (3.3–6.1)		11	1.2 (0.5–2.0)	
LET										
Total				<0.001			<0.001			<0.001
Negative	4,891	188	3.8 (3.3–4.4)		66	1.3 (1.0–1.7)		11	0.2 (0.1–0.4)	
Positive	573	163	28.4 (24.8–32.1)		143	25.0 (21.4–28.5)		52	9.1 (6.7–11.4)	
2017				<0.001			<0.001			<0.001
Negative	1,702	67	3.9 (3.0–4.9)		22	1.3 (0.8–1.8)		3	0.2 (0.0–0.4)	
Positive	256	72	28.1 (22.6–33.6)		49	19.1 (14.3–24.0)		16	6.3 (3.3–9.2)	
2018				<0.001			<0.001			<0.001
Negative	2,043	69	3.4 (2.6–4.2)		29	1.4 (0.9–1.9)		7	0.3 (0.1–0.6)	
Positive	200	58	29.0 (22.7–35.3)		61	30.5 (24.1–36.9)		24	12.0 (7.5–16.5)	
2019				<0.001			<0.001			<0.001
Negative	1,146	52	4.5 (3.3–5.7)		15	1.3 (0.7–2.0)		1	0.1 (0.0–0.3)	
Positive	117	33	28.2 (20.1–36.4)		33	28.2 (20.1–36.4)		12	10.3 (4.8–15.8)	
HIV				0.275			0.615			0.015
Negative	5,427	347	6.4 (5.7–7.0)		207	3.8 (3.3–4.3)		61	1.1 (0.8–1.4)	
Positive	37	4	10.8 (0.8–20.8)		2	5.4 (−1.9–12.7)		2	5.4 (−1.9–12.7)	

*CT, Chlamydia trachomatis; NG, Neisseria gonorrhoeae; HIV, Human Immunodeficiency Virus; CI, confidence interval; LET, leukocyte esterase test*.

### Prevalence of CT and NG Infections

Of 5,464 males, the prevalence of CT and NG were 6.4% (95% CI: 5.8–7.1%) and 3.8% (95% CI: 3.3–4.3%), respectively. 63 (1.2%, 95% CI: 0.9–1.4%) participants were co-infected. The annual CT prevalence were 7.1%, 5.7%, and 6.7% and the annual NG prevalence were 3.6%, 4.0%, and 3.8% from 2017 to 2019, respectively.

Among different age groups, the CT prevalence ranged from 8.3%(6.4–10.2%) (age ≤ 25 years) to 1.9%(0.4–3.4%) (age >65 years). A significant difference in age was observed for the CT infection rate (*P* < 0.001). Of the 209 NG-positive males, 34 (16.3%) were ≤ 25 years and had an NG positivity of 4.2% (95% CI: 2.8–5.5%). However, unlike CT prevalence, NG prevalence was not significantly associated with age (*P* = 0.289). Also, no significant difference in CT prevalence was observed according to geographic distribution (*P* = 0.746). However, the highest prevalence (4.7%) of NG infections was observed in the northern region, which was significantly higher than that in the Pearl River Delta region (2.9%, *P* = 0.01).

Among the 37 individuals living with HIV, the prevalence rate of CT was 10.8%, while the prevalence rate of NG was 5.4% and the prevalence rate of co-infection with CT and NG was 5.4%. Compared to the HIV-negative individuals, individuals living with HIV had a higher prevalence of CT, NG and co-infection with CT and NG, although only co-infection was statistically significant (*P* = 0.015).

### LET-Positive Urine Samples

Of 5,464 males, a total of 573 (10.5%) specimens showed a positive LET result. The positive rates of CT and NG in the LET-positive population were 28.4% and 25.0%, respectively. Fifty-two (9.1%) participants were NG and CT co-infected (Table 2).

**Table 2:** Characteristics of LET-positive patients in Guangdong, China, 2017–2019 (*N* = 5,464).

**Infection**	**NAAT (*****N*** **= 5,464)**		**LET+ (*****N*** **= 573)**	
	***N***	**%**	***n***	**%**
No infection	4,967	90.9	319	55.7
*Gonorrhoeae* or *chlamydia*	497	9.1	254	44.3
*Gonorrhoeae* and *chlamydia*	63	1.2	52	9.1
Only *gonorrhoeae*	146	2.7	91	15.9
Only *chlamydia*	288	5.3	111	19.4
Any *gonorrhoeae*	209	3.8	143	25.0
Any *chlamydia*	351	6.4	163	28.4

*NAAT, nucleic acid amplification test; LET, leukocyte esterase test. (Any chlamydia = Gonorrhoeae and chlamydia + Only chlamydia) (Any gonorrhoeae = Gonorrhoeae and chlamydia + Only gonorrhoeae)*.

### Diagnostic Performance of LET to Predict CT Infection

Among 573 LET-positive specimens, 163 had positive CT results. LET showed a sensitivity of 46.4% (95% CI: 41.2–51.7%), specificity of 92.0% (95% CI: 91.2–92.7%), PPV of 28.4% (95% CI: 24.8–32.1%), and NPV of 96.1% (95% CI: 95.6–96.7%) (Table 3). The LET 3+ result had a comparable specificity of 98.7% (χ^2^ = 106.871, *P* < 0.001), but the sensitivity was only 11.1% (95% CI: 7.8–14.4%), which was significantly lower than that for all LET-positive patients (χ^2^= 254.688, *P* < 0.001) (Table 3).

**Table 3:** Diagnostic performance of LET to predict *Chlamydia* infection.

	**Positive**	**Negative**	**Sensitivity (%)**	**Specificity (%)**	**PPV (%)**	**NPV (%)**
**Total**				
1+, 2+, 3+	163	410	46.4 (41.2–51.7)	92.0 (91.2–92.7)	28.4 (24.8–32.1)	96.1 (95.6–96.7)
0	188	4,703				
2+, 3+	93	198	26.5 (21.9–31.1)	96.1 (95.6–96.7)	32.0 (26.6–37.3)	95.0 (94.4–95.6)
0, 1+	258	4,915				
3+	39	69	11.1 (7.8–14.4)	98.7 (98.3–99.0)	36.1 (27.1–45.2)	94.2 (93.5–94.8)
0, 1+, 2+	312	5,044				
**HIV negative**						
1+, 2+, 3+	161	408	46.4 (41.2–51.6)	92.0 (91.2–92.7)	28.3 (24.6–32.0)	96.2 (95.6–96.7)
0	186	4,672				
2+, 3+	93	197	26.8 (22.1–31.5)	96.1 (95.6–96.7)	32.1 (26.7–37.4)	95.1 (94.5–95.6)
0, 1+	254	4,883				
3+	39	69	11.2 (7.9–14.6)	98.6 (98.3–99.0)	36.1 (27.1–45.2)	94.2 (93.6–94.8)
0, 1+, 2+	308	5,011				
**HIV positive**						
1+, 2+, 3+	2	2	50.0 (1.0–99.0)	93.9 (85.8–102.1)	50.0 (1.0–99.0)	93.9 (85.8–102.1)
0	2	31				
2+, 3+	0	1	0.0 (0.0–0.0)	97.0 (91.1–102.8)	0.0 (0.0–0.0)	88.9 (78.6–99.2)
0, 1+	4	32				
3+	0	0	0.0 (0.0–0.0)	100.0 (100.0–100.0)	–	89.2 (79.2–99.2)
0, 1+, 2+	4	33				

*LET, leukocyte esterase test; PPV, positive predictive value; NPV, negative predictive value*.

Among 573 LET-positive specimens, there were four specimens in the HIV-positive group and 569 in the HIV-negative group. Among HIV-positive individuals, LET showed a sensitivity of 50.0% (95% CI: 1.0–99.0%) and a specificity of 93.9% (95% CI: 85.5–102.1%). Among HIV-negative individuals, LET showed a sensitivity of 46.4% (95% CI: 41.2–51.6%) and a specificity of 92.0% (95% CI: 91.2–92.7%). Compared to the HIV-negative individuals, higher sensitivity and specificity were observed for HIV-positive individuals, but there was no statistical difference (*P* = 0.886 and *P* = 0.678) (Table 3).

### Diagnostic Performance of LET to Predict NG Infection

Among 573 LET-positive specimens, 143 had a positive result for NG. LET showed a sensitivity of 68.4% (95% CI: 62.1–74.7%), specificity of 91.8% (95% CI: 91.1–92.6%), PPV of 25.0% (95% CI: 21.4–28.5%), and NPV of 98.7% (95% CI: 98.3–99.0%) (Table 4). The LET 3+ result had a comparable specificity of 99.0% (95% CI: 98.7–99.3%, *P* < 0.001), but the sensitivity of 26.8% (95% CI: 20.8–32.8%) was significantly lower than that for all LET-positive patients (*P* < 0.001) (Table 4).

**Table 4:** Diagnostic performance of LET to predict *Gonorrhoeae* infection.

	**Positive**	**Negative**	**Sensitivity (%)**	**Specificity (%)**	**PPV (%)**	**NPV (%)**
**Total**						
1+, 2+, 3+	143	430	68.4 (62.1–74.7)	91.8 (91.1–92.6)	25.0 (21.4–28.5)	98.7 (98.3–99.0)
0	66	4,825				
2+, 3+	112	179	53.6 (46.8–60.3)	96.6 (96.1–97.1)	38.5 (32.9–44.1)	98.1 (97.8–98.5)
0, 1+	97	5,076				
3+	56	52	26.8 (20.8–32.8)	99.0 (98.7–99.3)	51.9 (42.4–61.3)	97.1 (96.7–97.6)
0, 1+, 2+	153	5,203				
**HIV negative**						
1+, 2+, 3+	141	428	68.1 (61.8–74.5)	91.8 (91.1–92.5)	24.8 (21.2–28.3)	98.6 (98.3–99.0)
0	66	4,792				
2+, 3+	112	178	54.1 (47.3–60.9)	96.6 (96.1–97.1)	38.6 (33.0–44.2)	98.2 (97.8–98.5)
0, 1+	95	5,042				
3+	56	52	27.1 (21.0–33.1)	99.0 (98.7–99.3)	51.9 (42.4–61.3)	97.2 (96.7–97.6)
0, 1+, 2+	151	5,168				
**HIV positive**						
1+, 2+, 3+	2	2	100.0 (100.0–100.0)	94.3 (86.6–102.0)	50.0 (1.0–99.0)	100.0 (100.0–100.0)
0	0	33				
2+, 3+	0	1	0.0 (0.0–0.0)	97.1 (91.6–102.7)	0.0 (0.0–0.0)	94.4 (87.0–101.9)
0, 1+	2	34				
3+	0	0	0.0 (0.0–0.0)	100.0 (100.0–100.0)	–	94.6 (87.3–101.9)
0, 1+, 2+	2	35				

*LET, leukocyte esterase test; PPV, positive predictive value; NPV, negative predictive value*.

LET showed a sensitivity of 100.0% (95% CI: 100.0–100.0%), specificity of 94.3% (95% CI: 86.6–102.0%), PPV of 50.0% (95% CI: 1.0–99.0%), and NPV of 100.0% (95% CI: 100.0–100.0%) among HIV-positive individuals. Although there was no significant statistical difference, higher sensitivity (*P* = 0.334) and specificity (*P* = 0.593) were found in HIV-positive individuals in terms of PPV(*P* = 0.245) and NPV(*P* = 0.500) when compared to HIV-negative individuals (Table 4).

### Diagnostic Performance of LET to Predict CT and NG Infection

Among 573 LET-positive specimens, 254 were positive for either CT or NG. The total LET-positive outcome showed that the sensitivity, specificity, PPV, and NPV were 51.1% (95% CI: 46.7–55.5%), 93.6% (95% CI: 92.9–94.3%), 44.3% (95% CI: 40.3–48.4%), and 95.0% (95% CI: 94.4–95.6%), respectively (Table 5). The LET 3+ result had a comparable specificity of 99.3% (95% CI: 99.1–99.5%; χ^2^ = 236.261, *P* < 0.001), but the sensitivity of 14.7% (95% CI: 11.6–17.8%) was significantly lower than that for all LET-positive patients (χ^2^ = 149.303, *P* < 0.001). LET showed a sensitivity of 50.0% (95% CI: 1.0–99.0%), specificity of 93.9% (95% CI: 85.5–102.1%), PPV of 50.0% (95% CI: 1.0–99.0%), and NPV of 93.9% (95% CI: 85.5–102.1%) among HIV-positive individuals (Table 5).

**Table 5:** Diagnostic performance of LET to predict *Chlamydia* or *Gonorrhoeae* infection.

	**Positive**	**Negative**	**Sensitivity (%)**	**Specificity (%)**	**PPV (%)**	**NPV (%)**
**Total**						
1+, 2+, 3+	254	319	51.1 (46.7–55.5)	93.6 (92.9–94.3)	44.3 (40.3–48.4)	95.0 (94.4–95.6)
0	243	4,648				
2+, 3+	166	125	33.4 (29.3–37.5)	97.5 (97.0–97.9)	57.0 (51.4–62.7)	93.6 (92.9–94.3)
0, 1+	331	4,842				
3+	73	35	14.7 (11.6–17.8)	99.3 (99.1–99.5)	67.6 (58.8–76.4)	92.1 (91.4–92.8)
0, 1+, 2+	424	4,932				
**HIV negative**						
1+, 2+, 3+	252	317	51.1 (46.7–55.5)	93.6 (92.9–94.3)	44.3 (40.2–48.4)	95.0 (94.4–95.6)
0	241	4,617				
2+, 3+	166	124	33.7 (29.5–37.8)	97.5 (97.1–97.9)	57.2 (51.5–62.9)	93.6 (93.0–94.3)
0, 1+	327	4,810				
3+	73	35	14.8 (11.7–17.9)	99.3 (99.1–99.5)	67.6 (58.8–76.4)	92.1 (91.4–92.8)
0, 1+, 2+	420	4,899				
**HIV positive**						
1+, 2+, 3+	2	2	50.0 (1.0–99.0)	93.9 (85.8–102.1)	50.0 (1.0–99.0)	93.9 (85.8–102.1)
0	2	31				
2+, 3+	0	1	0.0 (0.0–0.0)	97.0 (91.1–102.8)	0.0 (0.0–0.0)	88.9 (78.6–99.2)
0, 1+	4	32				
3+	0	0	0.0 (0.0–0.0)	100.0 (100.0–100.0)	–	89.2 (79.2–99.2)
0, 1+, 2+	4	33				

*LET, leukocyte esterase test; PPV, positive predictive value; NPV, negative predictive value*.

### Cost-Effective Evaluation

The total health provider economic cost (including start-up, test kits, staff time, overheads) for CT, NG and LET was $60,104, $60,104, and $7,103.2, respectively. The positive numbers for CT, NG and LET were 351, 209 and 573, respectively. The ICER using economic costs per additional person CT positive and NG positive was –$238.74 and –$145.60 compared with LET positive, respectively.

## Discussion

This study expanded the literature by evaluating the predictive value of LET for CT and NG infection. Our results demonstrate that LET has a high NPV for CT and NG infection under a comparatively high prevalence of CT and NG infection in Guangdong Province, China. Moreover, LET is a cost-effective test that can be used as a prescreening method for CT and NG infection.

In this study, we found comparatively high prevalence rates of CT and NG among males attending HIV/STI clinics in Guangdong Province at 6.4% and 3.8%, respectively. The prevalence of CT in our study is comparable to that of CT infection among men attending sexually transmitted infection clinics in Paris (5.7%) and US CDC (21, 22). Our findings were also in line with those of other studies conducted in China (23, 24). However, the prevalence of CT and NG in our study is lower compared to those described in western French Guiana and Brazil (25, 26). Prevalence rates may vary widely due to the characteristics of different study samples, including the age of the population, access to basic health services, levels of risky sexual behavior, and cultural traditions. A systematic literature review also found that social conditions, lack of economic opportunities, and risky sexual behavior are all closely associated with the incidence of STIs (27).

Our results showed that LET might have a comparable predictive value for CT and NG infection. Although LET showed moderate sensitivities of 46.4% and 68.4% for CT and NG, respectively, it had high specificity values of 92.0% and 91.8% and NPVs of 96.1% and 98.7%. This finding is in agreement with the results reported for the Central Australian Aboriginal population, which reported sensitivities of 60.0% and 66.7% and specificities of 94.7% and 90.7% compared with NAAT results for CT and NG detection, respectively (18). CT and NG can be accurately excluded if the LET result is negative. Therefore, LET would be valuable for predicting CT or NG infection in remote sites and clinics without NAAT. Though NAAT is the most sensitive test for CT and NG detection, it requires equipment and professional personnel to perform standardized specimen handling and the high cost of NAAT can also be an obstacle for implementation in low- and middle-income countries. Thus, the LET test as a simple and inexpensive method could be integrated into the standard of care for men attending HIV/STI clinics as prevention to inform clinical decisions for further evaluation and treatment of genital tract inflammation.

Besides, Seth C. Kalichman reported that 10% of sexually active men living with HIV who were currently receiving antiretroviral therapy tested positive for elevated urinary leukocyte esterase, which was consistent with our results. They found that men with elevated leukocyte activity reported significantly more risky sexual behavior in the previous 3-months, including more recent unprotected sexual intercourse (28). This finding reminds us again that LET test could be as a preventive measure in the care standards of male HIV/STI clinics and provide for further evaluation and behavioral interventions.

Mrus and colleagues reported that urine LET yielded the lowest incremental cost-effectiveness ratio in CT screening (29, 30). Compared with no screening, LET screening strategies reduced overall costs when the prevalence of chlamydial infection in males exceeded 2% (31). The ICER using economic costs per additional person CT positive and NG positive was –$238.74 and –$145.60 compared with LET positive, respectively, which were lower than that of NAAT detection. LET is a simple and inexpensive diagnostic test that can be used as a prescreening method for the detection of CT and NG infections in low- and middle-income countries.

Our results had several limitations, though. First, CT and NG were detected using urine samples. However, *Trichomonas vaginalis* and *Mycoplasma genitalium* are also common sexually transmitted diseases, which could also result in a positive LET result performed by urinalysis. The cost for each detection of CT, NG, *Trichomonas vaginalis* and *Mycoplasma genitalium* was approximately $11.00. If we could detect more pathogens, sensitivity might be improved. Second, the sensitivity and specificity of LET for predicting CT and NG infections are associated with the prevalence of CT and NG. In this study population, the prevalence rates of CT and NG were moderately high. Third, the number of HIV positive is small, and there may be a selection bias. Fourth, due to data limitations, our cost-effectiveness analysis results only used ICER for analysis about testing cost, we did not use a micro-costing approach to estimate the full economic cost from a health provider perspective and also not reported the tornado plot. However, even with these limitations, CT and NG were mostly accurately excluded when the LET result was negative; therefore, LET would be valuable for predicting CT and NG infections.

LET detection is a simple and inexpensive diagnostic test that is readily available at attending HIV/STI clinics. Though LET is not a diagnostic test for CT and NG infection, LET had a comparable high NPV value for the CT and NG infection. LET would be valuable for predicting CT and NG infection in low- and middle-income countries.

## Conclusions

In this study, LET is a cost-effective test and will be valuable for predicting CT and NG infection with a comparatively high prevalence in low- and middle-income countries.

## Data Availability Statement

The raw data supporting the conclusions of this article will be made available by the authors, without undue reservation.

## Ethics Statement

The studies involving human participants were reviewed and approved by the Ethics Committee of the Dermatology Hospital of Southern Medical University. Written informed consent to participate in this study was provided by the participants' legal guardian/next of kin.

## Author Contributions

XY and PZ performed data analysis in this study and jointly wrote the draft manuscript. ZM, WC, and QX performed NAAT experiments. ZhiqW, XLu, ZhizW, and XLi were responsible for the initial data collection. QW sorted data and compiled table preparation in this study. HZ and YX had the leading contribution to the design of studies and interpretation of the whole dataset. All authors read and approved the manuscript.

## Funding

This work was supported by the National Natural Science Foundation of China (81974307), the Natural Science Foundation of Guangdong Province (2018A030313662 and 2019A1515011827), the Research Foundation of Department of Education of Guangdong Province (2021KTSCX014), and Research Foundation of Traditional Chinese Medicine Bureau of Guangdong Province (20211276).

## Conflict of Interest

The authors declare that the research was conducted in the absence of any commercial or financial relationships that could be construed as a potential conflict of interest.

## Publisher's Note

All claims expressed in this article are solely those of the authors and do not necessarily represent those of their affiliated organizations, or those of the publisher, the editors and the reviewers. Any product that may be evaluated in this article, or claim that may be made by its manufacturer, is not guaranteed or endorsed by the publisher.
